# Mitigating coherent loss in superconducting circuits using molecular self-assembled monolayers

**DOI:** 10.1038/s41598-024-77227-7

**Published:** 2024-11-09

**Authors:** Mohammed Alghadeer, Archan Banerjee, Kyunghoon Lee, Hussein Hussein, Hossein Fariborzi, Saleem Rao

**Affiliations:** 1 Department of Physics, University of California, Berkeley, CA 94720 USA; 2 Applied Mathematics and Computational Research Division, Lawrence Berkeley National Laboratory, Berkeley, CA 94720 USA; 3 Materials Science Division, Lawrence Berkeley National Laboratory, Berkeley, CA 94720 USA; 4 CEMSE Division, King Abdullah University of Science and Technology, Thuwal, 23955 Saudi Arabia; 5 Department of Physics, King Fahd University of Petroleum and Minerals, Dhahran, 31261 Saudi Arabia; 6 Present Address: Department of Physics, Clarendon Laboratory, University of Oxford, Oxford, OX1 3PU UK; 7 Present Address: Department of Mechanical Engineering, MSFEA, American University of Beirut, Beirut, 1107 2020 Lebanon

**Keywords:** Surfaces, interfaces and thin films, Qubits

## Abstract

In planar superconducting circuits, decoherence due to materials imperfections, especially two-level-system (TLS) defects at different interfaces, is a primary hurdle for advancing quantum computing and sensing applications. Traditional methods for mitigating TLS loss, such as etching oxide layers at metal and substrate interfaces, have proven to be inadequate due to the persistent challenge of oxide regrowth. In this work, we introduce a novel approach that employs molecular self-assembled monolayers (SAMs) to chemically bind at different interfaces of superconducting circuits. This technique is specifically tested here on coplanar waveguide (CPW) resonators, in which this method not only impedes oxide regrowth after surface etching but can also tailors the dielectric properties at different resonators interfaces. The deployment of SAMs results in a consistent improvement in the measured quality factors across multiple resonators, surpassing those with only oxide-etched resonators. The efficiency of our approach is supported by microwave measurements of multiple devices conducted at millikelvin temperatures and correlated with detailed X-ray photoelectron spectroscopy (XPS) and transmission electron microscopy (TEM) characterizations of SAM-passivated resonators. The compatibility of SAMs materials with the established fabrication techniques offers a promising route to improve the performance of superconducting quantum devices.

## Introduction

Superconducting circuits serve as key components in constructing circuit quantum electrodynamics (cQED) systems. Over the past two decades and among different platforms for quantum computing, superconducting qubits have emerged as a promising avenue due to improvements in coherence times and gate fidelities^1–3^. Qubits are nonlinear LC oscillators and form the basic units of quantum information, while resonators are harmonic LC oscillators used mainly for addressing the states of qubits^4,5^, qubit-qubit coupling^6,7^ and quantum memory^8,9^. In practical devices, qubits and resonators are typically coupled to each other, forming a complex network of interaction that is crucial for performing quantum information processing. However, the construction of large and functional quantum devices necessitates substantial improvements in qubit relaxation and coherence times, which are currently orders of magnitude shorter than the thresholds imposed by constituent materials. At millikelvin temperatures and single-photon power excitations, materials loss in both qubits and resonators are predominantly attributed to a photon in the system being frequently coupled to a two-level system (TLS) that can exist inside bulk materials and at different interfaces, which is currently more prominently attributed to the presence of oxides at air-interfaces of metal and substrate^10^. Oxides related TLS loss are therefore recognized as a significant decoherence channel in quantum circuits, impacting both small features of Josephson junctions and large features of circuits’ capacitors and inductors of both qubits and resonators^10–12^.

Central to numerous superconducting qubit architectures, coplanar waveguide (CPW) resonators are pivotal components that also serve as standalone tools for characterizing materials’ loss under microwave single-photon excitations^10,13,14^. Efforts have been directed toward modifying resonator geometries^12,15^ and trying different materials^16–18^ to comprehend and diminish TLS loss that can exist in metal-air (MA), metal-substrate (MS), and substrate-air (SA) interfaces. Despite ongoing debates about the origin of TLS loss^11^, it is currently believed that such deleterious systems primarily stem from defects at these three interfaces^19,20^. It is also suspected that TLS loss might be present at interfaces containing chemical residues from the fabrication process and other sources^11,15^. Defects tend to manifest mainly in disordered, both structurally and chemically, materials at air-interfaces and efforts have been undertaken to comprehend and mitigate these issues^11,21^. While the MS interface can be ameliorated through substrate cleaning and improved epitaxial film growth^16,22–24^, addressing oxide growth at air-interfaces proves to be more challenging due to inherent materials properties of these amorphous layers. A previous report demonstrated a substantial improvement in resonators’ quality factors through chemically etching oxides present at MA and SA interfaces^25^. Nonetheless, the reformation of oxides after etching remains a persistent challenge for sustaining the improved quality factors. Approaches like self-limiting oxide growth^26^, nitrogen plasma treatments^27^ and surface encapsulation^28^ offer potential solutions for oxides regrowth. Recently, we presented preliminary outcomes related to the absorption of Octatrichlorosilane (OTS) self-assembled monolayers on Si and Nb surfaces, exploring their impact on oxide regrowth^29^. In this article, we delve into a detailed study involving other types of SAM materials absorbed on metal and substrate surfaces, investigating their role in suppressing oxide regrowth and improving the dielectric constants of MA and SA interfaces.

The ability to form a wide range of molecular SAMs on metallic/semiconducting surfaces through various processes, such as from a dip into evaporation-based deposition, is an attractive proposition^30,31^. Over the last three decades, SAMs absorbed onto diverse metallic and semiconducting surfaces have allowed to finely tune the physical and chemical properties of various interfaces^32–35^. This extends to the realms of molecular, organic, and conventional electronics, where SAMs play roles as electronic components and in controlling surface attributes such as work function and dielectric properties^35^. The suppression of oxide growth on semiconducting metal and oxide surfaces through SAMs absorption is a well-explored phenomenon^36,37^. Given that oxides present at various air-interfaces harbor structural and chemical defects, they are a major source of decoherence in quantum devices^11,19,20^, as these oxides foster TLS defects. SAMs at MA- and SA-interfaces can replace these defects with materials devoid of chemical and structural imperfections. Consequently, SAMs not only impede oxide regrowth but also substantially reduce undesired energy absorption in these additional layers due to their well-established defect-free structural and electronic characteristics^38,39^. The robust chemical bonding of SAMs at air-interfaces, determined by the binding ends of SAMs with the metal and substrate chemical structure, defines the crystal structure of the bonded SAM material, a phenomenon well-documented across various interfaces^30,31^. Defect-free SAM assembly on a smooth surface possesses the ability to minimize TLS loss to a very low level. Moreover, the dielectric constant of oxides at different device interfaces plays a crucial role in TLS loss mechanisms, and SAMs can be harnessed to control such loss since these materials have been demonstrated to modify surface dielectric properties, such as within a broad range of 2–20 dielectric constants^40–42^.

Recent theoretical and experimental results have demonstrated SAM-based modification of the dielectric constants of semiconductor/metal-oxide surfaces^41^, although studies focusing on loss tangents for such SAM-modified surfaces remain unexplored. Similar to recent reports^42^, superconducting CPW resonators provide a suitable platform for studying the dielectric constant and loss tangent of SAMs. Notably, SAMs materials are highly compatible with many established micro-/nano-fabrication processes^43^, making these materials feasible to be used at various stages of the fabrication process of superconducting devices. Additionally, the incorporation of SAM-based sandwich structures, featuring conducting and non-conducting SAMs, is commonplace in molecular electronics^44,45^. Thus, in addition to reducing surface losses in superconducting devices, SAMs could potentially be employed as well to mitigate losses in the fabrication of Josephson junctions when combined with a suitable insulating SAM layer.

In this work, we show the passivation of CPW resonators using molecular self-assembled monolayers on Nb and Si surfaces, including MA- and SA-interfaces, which results in a sustained improvement over time in the quality factors of multiple treated devices. This observation is supported by analytical scanning transmission electron microscopy (STEM), X-ray photoemission spectroscopy (XPS) and atomic force microscopy (AFM) measurements, in which all of these measurements collectively indicate the mitigation of TLS loss as a result of the uniform SAMs passivation of metal and substrate surfaces.

## Results and discussion

**Figure 1:**
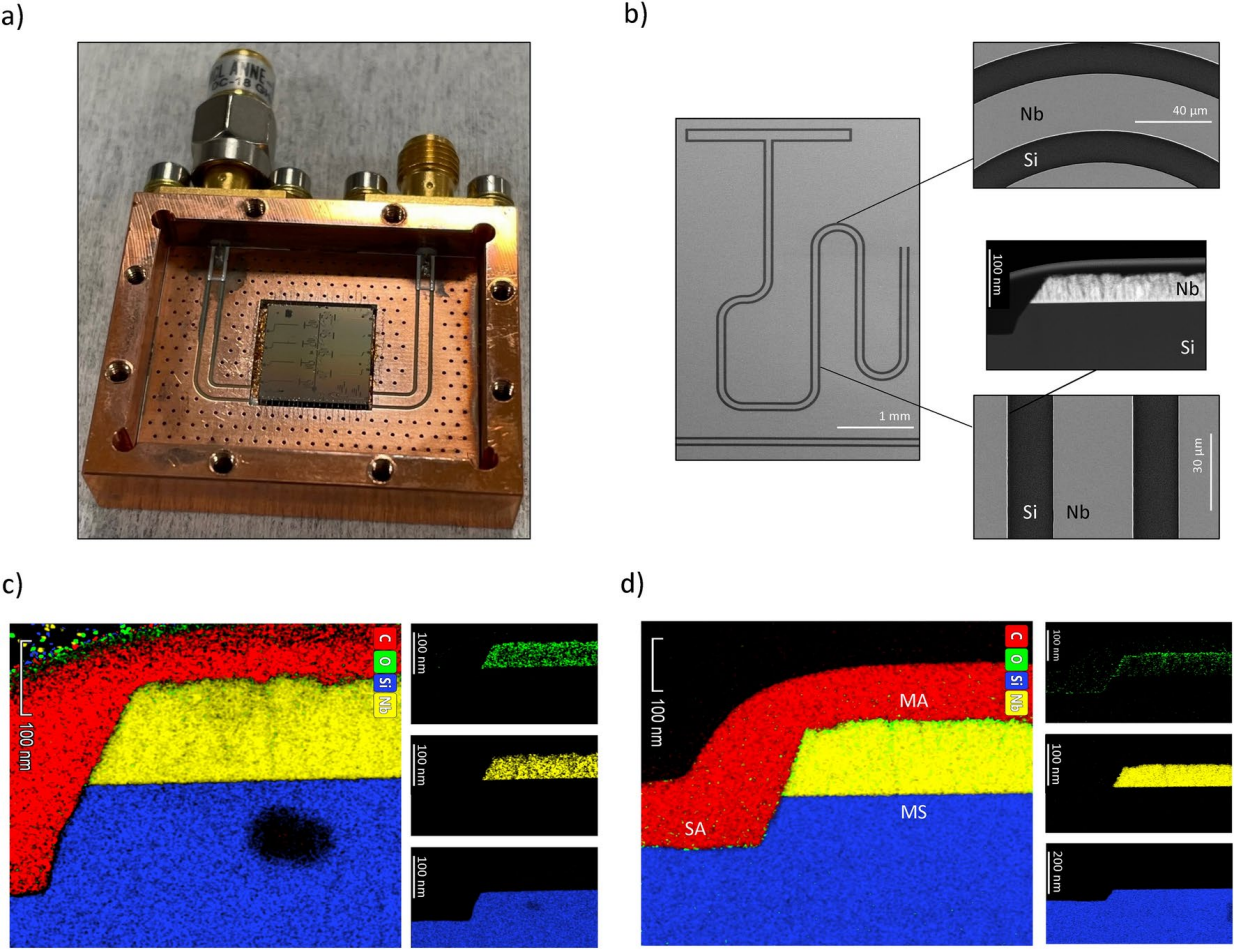
Resonators device and EDS colored STEM images of MA, MS and SA interfaces $\sim$ 20 days after fabrication and SAM treatment. (**a**) A 10 mm $\times$ 10 mm chip of resonators device in a sample box, featuring ten Nb CPW patterns on a Si substrate. (**b**) SEM images of a single resonator indicating the locations from which TEM cross-sectional lamella were extracted. Cross-sectional EDS-STEM images of different interfaces after post-fabrication BOE etching of MA and SA interfaces in which (**c**) is a sample without SAM treatment and (**d**) shows a treated sample with a double-layered SAMs adsorption of both OTS and MPA.

Device design and the STEM images are shown in Fig.  1a,b, respectively, illustrating CPW structures and the targeted areas for materials analysis. The cross-sectional STEM images (Fig.  1c,d) are particularly revealing, highlighting the stark contrast between untreated and SAM-treated surfaces in which this provides intricate nanometer-resolution EDS fluorescence mappings of the impact of self-assembled monolayers on oxide growth through illustrating elemental compositions and chemical states of the three resonator interfaces (MA, SA, and MS). In the untreated sample (Fig. 1c), a pronounced contrast of oxides at the MA interfaces is clearly present, consistent with expected oxide regrowth after post-fabrication BOE etching when the sample is exposed to ambient environment. These oxides at device interfaces are what to contribute significantly to TLS loss. In contrast, the SAM-treated sample after BOE etching (Fig.  1d) shows a markedly reduced oxide presence and more color-contrasted Nb and Si signals. This observation in STEM-EDS mappings shows elemental composition and provides an illustration of the reduced oxide growth on SAM-treated MA and SA interfaces.

Remarkably, our high-resolution STEM-EDS images also indicate that the grown layer of oxide on the MA interface has the ability to dissipate through the metal surface, impacting not only the MA interface but also the bulk dielectrics. Whereas in the crystalline material of Si substrate, oxides are not observed to be present inside the bulk material but only at the SA interface. This observation is clearly shown in Fig.  1c of the untreated sample, whereas the SAM-treated sample in Fig.  1d indicates significantly less amount of oxides both at the MA interface and inside the bulk material of Nb. The reason behind this observation is anticipated to be that SAM binds through a layer of oxygen atoms to the metal surface, which implies that there are no additional oxides except at the binding ends of the specific SAM molecule, which prevents further dissipation of oxide formation from the interface down to the bulk material. Our specific case necessitates further in-depth investigation into the concurrent binding of the double-layered OTS-MPA onto the Nb and Si surfaces, and how this dual-binding of SAMs molecules influences oxide penetration on the Nb thin film. In addition, the temporal aspect of these observations is critical as well, in which these imaging data were collected $\sim$ 20 days post-sample fabrication and preparation, a period sufficiently long for oxides to typically form. This time-dependent analysis is pivotal, as it confirms the lasting effectiveness of SAM treatment against oxide formation, a process that usually occurs rapidly in untreated conditions.

**Figure 2:**
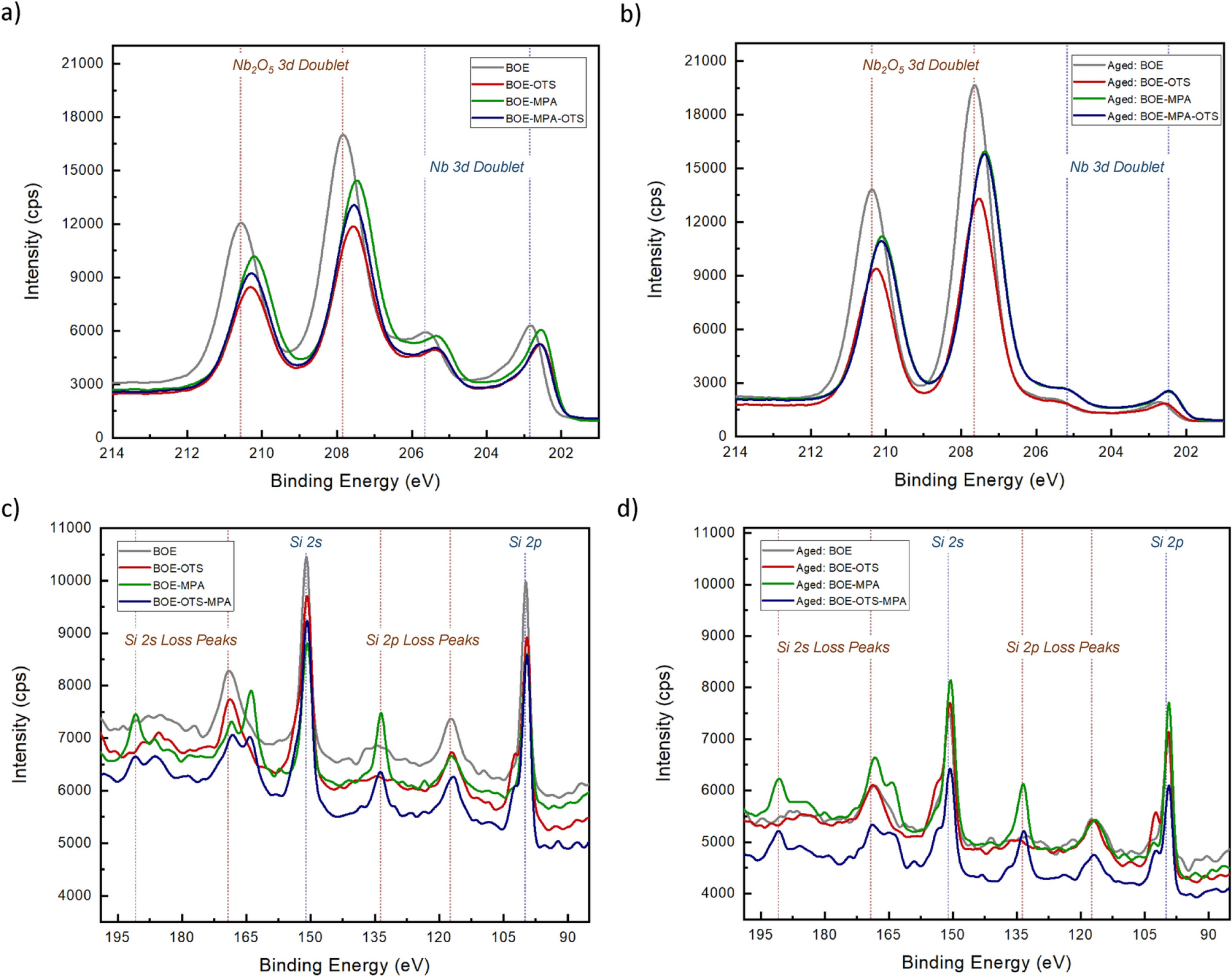
XPS analysis of Si and Nb surfaces after BOE etching and SAM passivation with OTS, MPA and double-layered OTS-MPA. XPS spectra of Nb surfaces for (**a**) fresh devices and (**b**) aged devices. XPS spectra of Si surfaces for (**c**) fresh devices and (**d**) aged devices. Even after $\sim$ 20 days, the amount of Nb and Si oxides in the SAMs treated samples is observed to be lower than the samples only etched with BOE.

Additionally, XPS measurements are shown in Fig.  2 obtained for Si and Nb air-interfaces subjected to different surface treatments, both for BOE etched resonators and for various SAMs treated resonators. Subsequent to BOE etching, all SAMs treated samples were promptly passivated with SAMs materials, with the detailed process described in the methods section.^29^. We obtained XPS data twice for each sample including for both fresh devices and for aged devices after $\sim$ 20 days of sample preparation and before XPS measurements in order to validate the effectiveness of SAMs surface treatments over time. Moreover, XPS measurements of Nb surfaces (Fig.  1a,b) exhibit a more pronounced suppression of Nb oxide peaks in the spectra of SAM passivated samples as compared to samples solely etched with BOE, both in the case of fresh and aged samples. Both OTS and MPA have proven effective in restraining oxide regrowth on Nb after sufficient BOE etching. These SAMs molecules have a propensity to bind at minimum oxidized surfaces^35^ with extensive validation for SAM bonding on materials like silicon, copper, aluminum and palladium, in comparison to niobium^36,37^. Remarkably, the simultaneous adsorption of double-layered OTS-MPA on Nb has shown to be effective, while the maximal suppression of Nb oxide peaks happened in the treatment with OTS for both cases of fresh and aged devices.

In the case of Si XPS spectra (Fig.  1c,d), both Si 2s and 2p loss peaks were attenuated following SAMs treatments in the case of fresh and aged samples. Notably, Si 2s and 2p peaks exhibited maximal intensity in the case of fresh BOE etched sample (Fig.  1c), yet these peaks significantly decreased in the case of the aged BOE etched sample due to oxide growth on Si surface (Fig.  1d). Furthermore, XPS data demonstrated a reduction in Si loss peaks attributed to energy loss (plasmon) effects subsequent to BOE cleaning. In stark contrast, SAM passivation led to a further reduction in plasmon peaks for both fresh and aged samples. The profile of Si 2s and 2p plasmon loss peaks is influenced by the materials traversed by the photoelectrons, and these loss structures can potentially interfere with the interpretation and quantification of other spectral peaks, such as the Si 2s and 2p signals in this context.

**Figure 3:**
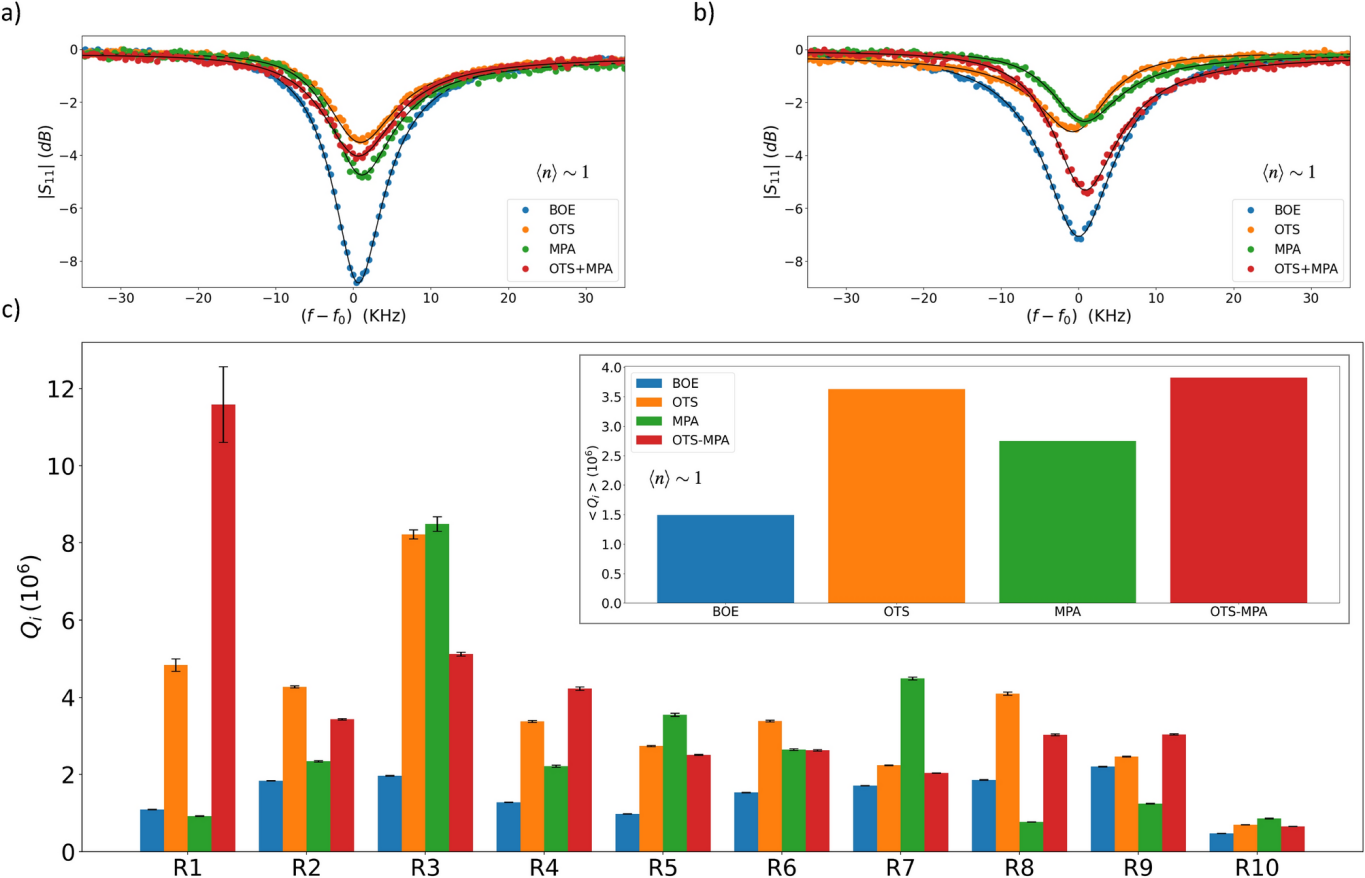
Microwave reflectometry measurements comparing the performance of resonators with different surface treatments. Measurements of single resonators in fresh BOE-etched and SAM-passivated samples are shown in (**a**), while aged BOE-etched and SAM-passivated samples are shown in (**b**). Detailed resonators $Q_{i}$ measurements across each fresh device is shown in (**c**) with average $Q_{i}$ over each device shown in the inset figure. All measurements were conducted at single-photon input power. Both OTS and double-layered OTS-MPA treatments achieved comparable high average $Q_{i}$ across multiple resonators.

**Figure 4:**
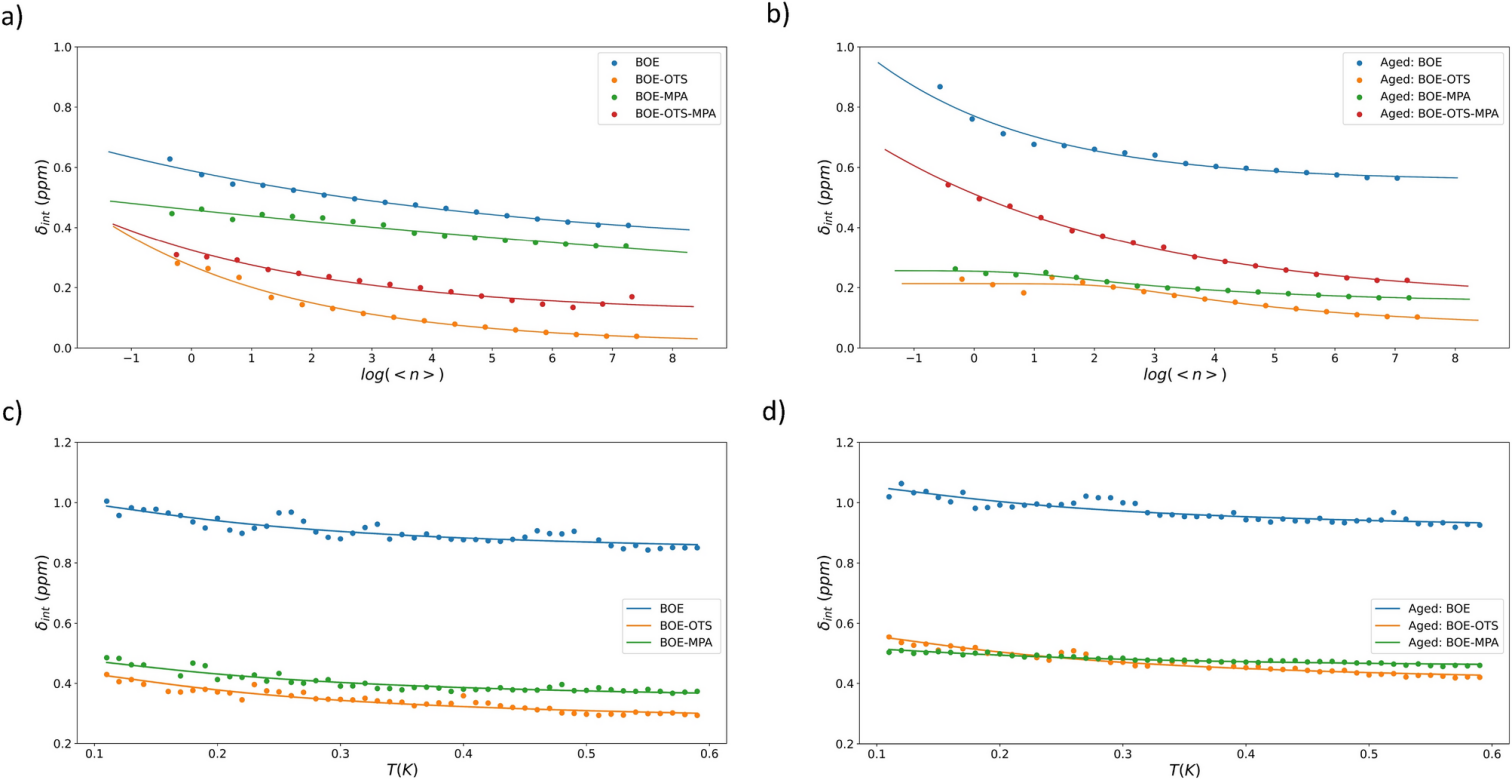
Power and Temperature dependent measurements of resonators in multiple device with different surface treatments. Resonator intrinsic loss $\delta _{\text {in}}$ as function of average photon number for fresh and aged devices in (**a**) and (**b**), respectively. Resonator intrinsic loss $\delta _{\text {in}}$ as function of temperature for fresh and aged devices in (**c**) and (**d**). For both fresh and aged devices, OTS-treated resonators show minimal intrinsic loss.

In this work, $Q_{i}$ data was measured for ten CPW resonators in multiple devices for different surface treatments. Detailed microwave measurements at single-photon regime are shown in Fig. 3 for various cases. Microwave signals for fresh and aged devices are shown in Fig. 4a,b, respectively, where the decreased peak’s depth at resonance indicates reduced loss at MA and SA interfaces due to SAMs passivation. In both cases, SAMs treated resonators indicate less transmission loss in the spectrum of measured signal. Detailed $Q_{i}$ measurements across all resonators for multiple freshly treated devices are shown in Fig. 4c, in which $Q_{i}$ values for SAMs treated resonators surpass most of only BOE-etched resonators. The average $Q_{i}$ across each device indicate resonators treated with both OTS and double-layered OTS-MPA achieved more than a factor of two improvement over multiple resonators. Furthermore, the observed improvement in $Q_i$ due to SAMs treatments is indicative of a substantial reduction in the dielectric loss due to mitigation of TLS loss at MA and SA interfaces. The data suggests that the molecular layers introduced by the SAM treatments create a more favorable interface for the resonator, thus enhancing the coherence and reducing the energy dissipation in these systems.

The power- and temperature- dependent results in Fig. 4 offer comprehensive measurements of resonators intrinsic loss, denoted as $\delta _{\text {int}}$ , across a spectrum of operational conditions for devices with various surface treatments. Panels (a) and (b) delineate the dependency of on the average photon number $\langle n \rangle$ for fresh and aged devices, respectively. It is evident that in both temporal states, the resonators that have undergone SAM treatment manifest a markedly lower intrinsic loss compared to those subjected solely to BOE etching . This discrepancy in loss is particularly pronounced at low photon numbers, suggesting that the SAMs passivation serves as an effective barrier, mitigating the impact of TLS loss, which are known to be predominant at these energy scales. The consistency of this effect across both fresh and aged devices shows the robustness of SAMs surface treatments in preserving the integrity of the resonator surfaces over time.

Moving to the temperature dependency illustrated in panels (c) and (d), we observe a similar trend wherein the SAMs treated resonators maintain a lower $\delta _{\text {int}}$ across the measured temperature range. The implication here is twofold: firstly, that the surface treatments have a persistent effect regardless of thermal fluctuations on these resonators, and secondly, that the intrinsic loss $\delta _{\text {int}}$ is intimately connected to TLS loss $\delta _{\text {TLS}}$, which is both a power- and temperature-dependent mechanism in superconducting circuits. The thermal stability of the SAMs treated resonators suggests that the surface passivation provided by both OTS and MPA treatments is effective in curtailing the TLS-related dissipation mechanisms, which in some cases result in increased dielectric loss at higher temperatures. This is of a particular interest for quantum computing applications, where thermal stability is a crucial factor for the operation of superconducting qubits. These results collectively highlight the efficacy of SAMs surface treatments in mitigating TLS loss, which is a significant step forward in the fabrication and long-term reliability of superconducting quantum circuits.

## Conclusion

In conclusion, our comprehensive investigation indicates the impact of self-assembled monolayers on mitigating oxide growth on interfaces of superconducting resonators, directly correlating with the enhanced device performance due to reduced interface loss. High-resolution STEM-EDS and XPS results reveal that SAM treatments significantly suppress oxide formation at metal-air and substrate-air interfaces, even in ambient conditions and over prolonged periods. This suppression is notably evident in the stark contrast observed in oxide presence between untreated and SAM-treated samples, with the latter showcasing reduced oxide growth on Nb and Si surfaces. These findings are further substantiated by microwave measurements, which indicate a substantial improvement in the quality factors of SAM-treated resonators, surpassing those only subjected to BOE etching. Power- and temperature-dependent measurements show the robustness of SAM treatments in maintaining lower intrinsic loss across a spectrum of operational conditions, illustrating the potential of SAMs passivation in enhancing the coherence of quantum devices. Our work not only provides a pathway towards mitigating TLS loss in superconducting quantum circuits but also sets a benchmark for future investigations into the optimization of surface treatments in fabricating sustained and scalable quantum devices.

## Methods

### Fabrication process

Resonators samples were fabricated by a process that includes e-beam lithography of $\text {Nb}$ film deposited on polished high-resistivity ($> 8000 \, \Omega \text {-cm}$) $\text {Si}$ wafer. One resonators device consists of ten CPW resonators capacitively coupled to a $50 \, \Omega$ transmission line with gap and center trace width of $10 \, \mu \text {m}$ and $20 \, \mu \text {m}$, respectively. First, the $\text {Si}$ wafer was cleaned for 10 minutes in piranha solution made by a mixture of sulfuric acid and hydrogen peroxide at $120 \, ^{\circ }\text {C}$, and then followed by one minute cleaning in 49% Hydrofluoric acid (HF) in order to remove contaminants and native oxide on $\text {Si}$ surface. The wafer was then inserted into an ultra-high vacuum load-locked sputter deposition system immediately after cleaning, with the process chamber base at a pressure of $< 5 \times 10^{-8}$ Torr. Niobium film of $\sim 180 \, \text {nm}$ was then deposited by magnetron sputtering at room temperature and at deposition pressure of $1.5 \, \text {mTorr}$ for 10 minutes. Following that, MicroChem MMA EL-13 copolymer resist was exposed to define CPW patterns using a 100 kV Raith Electron Beam Pattern Generator (EBPG 5150). After writing CPW patterns, the wafer was developed in a 3:1 mixture of IPA:MIBK solution at room temperature before starting the dry etching process in an inductively coupled reactive ion etcher ($\text {BCl}_3/\text {Cl}_2$ process). Finally, residual resist was cleaned using Microposit Remover N-Methyl-2-pyrrolidone (NMP) 1165 in a water bath at $80 \, ^{\circ }\text {C}$ for $\sim 2$ hours, and then the wafer was coated with resist and diced. Dicing resist on each chip was removed with hot NMP in a water bath at $80 \, ^{\circ }\text {C}$ for $\sim 12$ hours.

**Figure 5:**
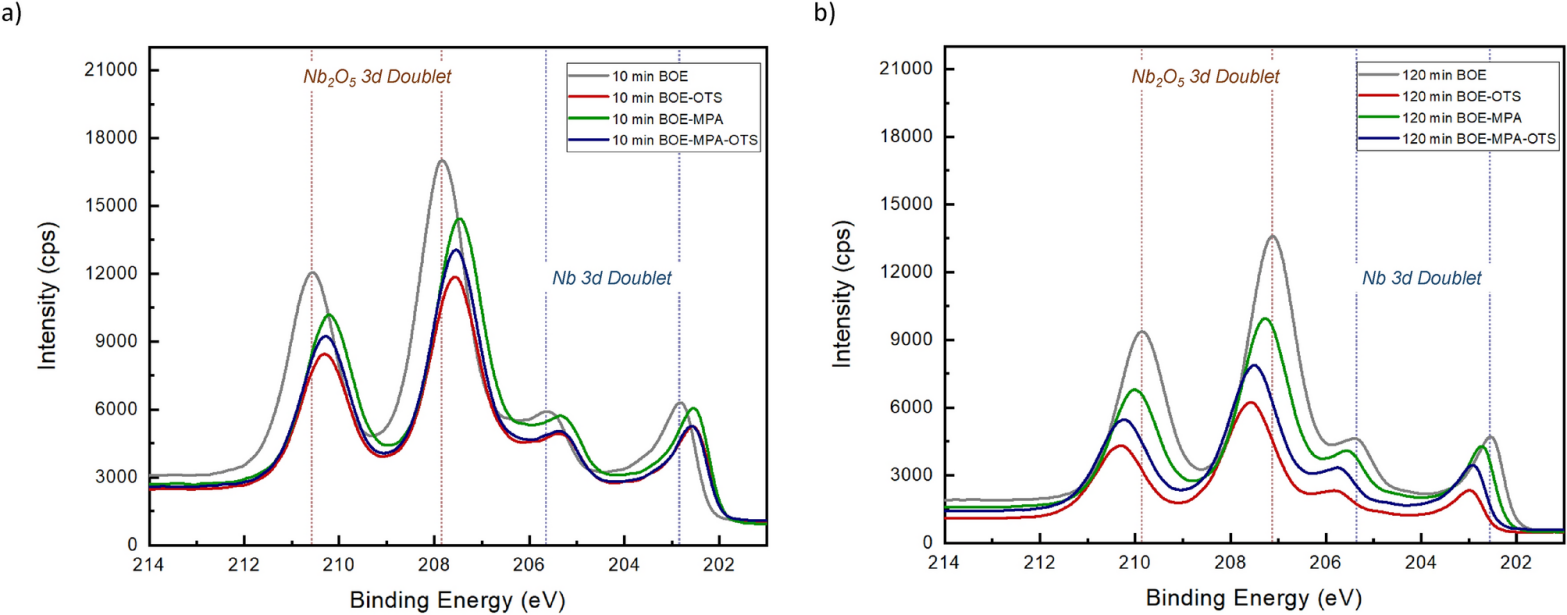
XPS analysis of Nb surface for 10 min (**a**) and 120 min (**b**) BOE etching followed by SAM passivation with OTS, MPA and double-layered OTS-MPA. For both cases, SAM passivation is consistent on Nb, suggesting the compatibility of this surface treatments with BOE etching.

Post-fabrication process includes etching of metal and substrate surfaces using 5:1 BOE to clean processing oxides and then immediately passivating the sample using dipping method for SAM assembly on Nb and Si air-interfaces. Specifically, for all samples in this study, the post-fabrication process includes cleaning the dicing resist in NMP as described above, followed by 20 minutes cleaning with BOE and DI water rinse in three successive beakers^25^. We then used a well-reported process in the literature^29,30,46^ in which resonator samples were dipped in a 0.5 mM toluene solution for octatrichlorosilane (OTS) SAM adsorption to achieve SAM coverage of both Nb and Si air-interfaces for an optimized time interval of $\sim$ 2.0 min. Similarly, for 2-merceptoimdazole (MPA) adsorption, resonator samples were dipped in a 1.0 mM ethanol solution of MPA for $\sim$ 5.0 min. For a double-layered SAMs adsorption of both OTS and MPA which involves first dipping in 0.5 mM toluene solution of OTS for $\sim$ 2.0 min followed by a second dip in 1.0 mM ethanol solution of MPA for $\sim$ 5.0 min. We observed consistent passivation with SAM materials with different BOE etching time intervals as shown in Fig. 5. For material analysis, XPS data, AFM and STEM images were collected and analyzed for fresh samples and other samples were prepared using the same process and were cooled down for microwave measurements. In addition, the design and geometric parameters of our CPW resonators are given in Table 1.

**Table 1:** Geometric design parameters of a CPW resonator.

Geometric parameter	Symbol	Standard value
CPW gap width	*w*	10 $\upmu$m
Central line width	*s*	20 $\upmu$m
Nb thin film thickness	*t*	180 nm
Si substrate thickness	*h*	674 $\upmu$m

### Materials analysis

Transmission electron microscopy (TEM) cross-sectional lamella was prepared with dual-beam Thermo Scientific Helios G4 SEM and Focused Ion Beam (FIB) microscope, equipped with a field emission gun. To protect the region of interest, first we deposited an electron-beam induced carbon layer, and then electron-beam induced platinum and ion-beam induced platinum layers. Transmission electron microscopy (TEM) and Scanning TEM (STEM) investigations were performed with a double Cs-Corrected Thermo Scientific Titan Themis-Z microscope equipped with a field emission gun operating at 300 kV, a Wien type monochromator, a Super-X system for X-ray energy dispersive spectroscopy (EDS), a Thermo ScientificT Ceta camera 16 M, and a Gatan Quantum ER energy filter.

Scanning electron microscope (SEM) images were acquired using a Thermo Scientific Quattro Environmental Scanning Electron Microscope and a ZEISS Gemini ULTRA-55 Field Emission Scanning Electron Microscope. High magnification images were collected at 2 kV through a high efficiency In-lens Secondary Electrons detector. Low magnification, large field of view images, used 5 kV and a conventional Everhart Thornley Secondary Electron Detector.

X-ray photoemission spectroscopy (XPS) experiments were performed on a Kratos Axis SUPRA instrument equipped with a monochromatic Al K$\alpha$ X-ray source (h$\upsilon$ = 1486.6 eV) operated at 75 W under UHV conditions ($\sim$ 10-9 mbar). The spectra were recorded in a hybrid mode using electrostatic and magnetic lenses and an aperture slot of 300 by 700 $\upmu$m. The survey and high-resolution spectra were acquired at fixed analyzer pass energies of 80 eV and 20 eV, respectively. The samples were mounted in a floating mode to avoid differential charging and thus the spectra were acquired under charge neutralization conditions.

**Figure 6:**
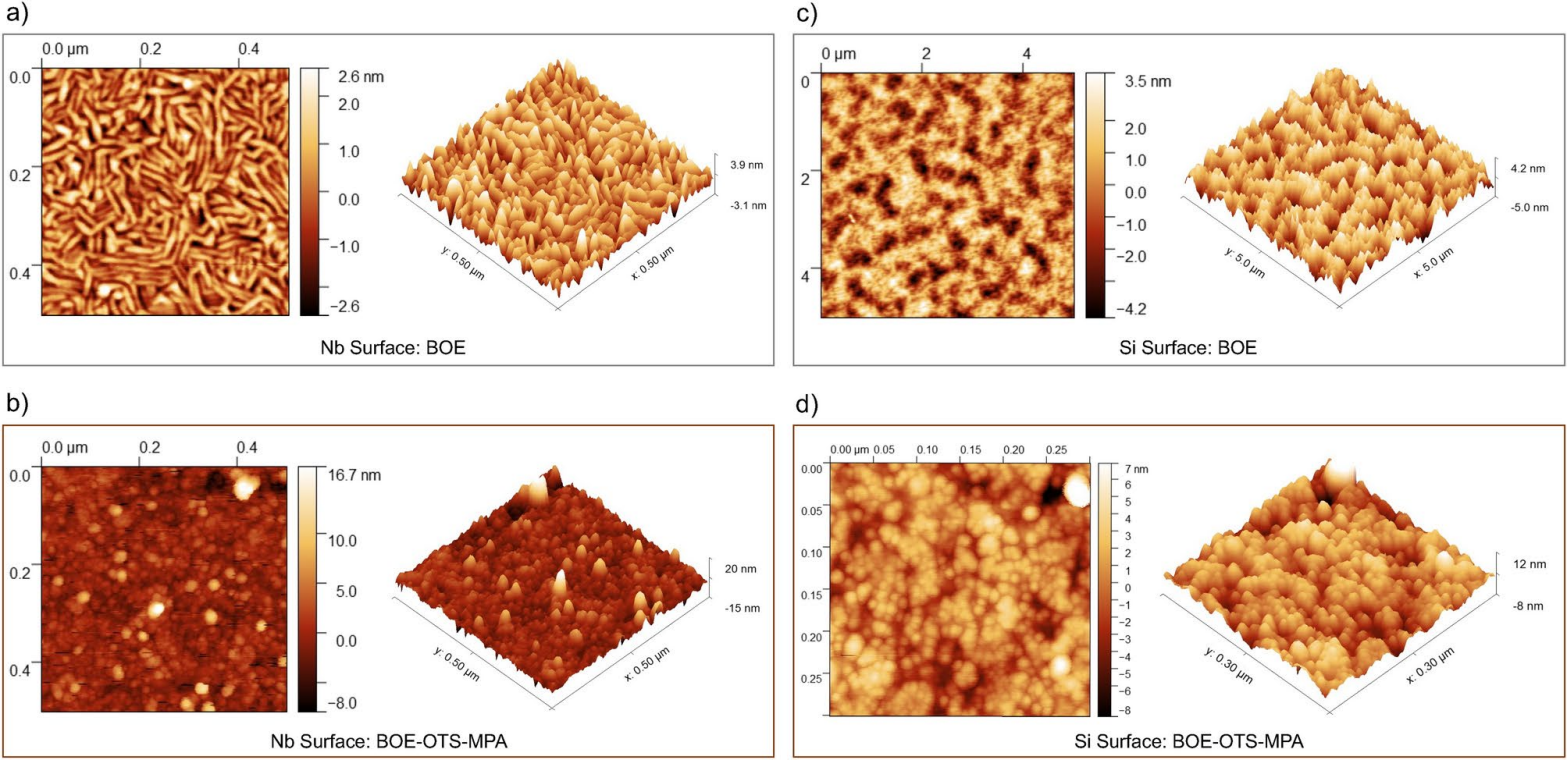
AFM imaging of Nb and Si surfaces after BOE etching and followed by SAMs treatments. Nb surface after BOE etching (**a**) and followed by a double-layered OTS-MPA in (**b**). Si surface after BOE etching (**c**) and followed by a double-layered OTS-MPA in (**d**). For both Nb and Si surfaces, SAMs passivation is uniform in the imaged surfaces, though the scale varies slightly between the Nb and Si images to capture the relevant surface features clearly.

Atomic force microscopy (AFM) in both tapping and non-contact modes was employed to image surface topography and mechanical properties of SAM passivated samples as shown in Fig. 6 for both Nb and Si surfaces. Cypher AFM system was used to images the samples and the analysis was enhanced by the precision of a Budget Sensor Tap300-G AFM tip, which operates at 300 kHz, and a Nanosensors Ultra Short Cantilever USC-F1.2 operating at 1.2 MHz. AFM image of the BOE-etched surface is comparable to previously reported results^47^. However, the surface roughness of our films is higher, likely due to the BOE etching process. This increased roughness is also observed on the BOE-etched Si surface. Ideally, SAMs form perfect monolayers at air interfaces upon deposition^30^. This perfection depends on several factors, including surface smoothness, SAM-substrate bonding strength, the type of SAM itself, are few of them because SAM assembly can very a complex process^30,31^. Upon immersion in a SAM solution, the anchoring group of the SAM molecule rapidly binds to the surface (within seconds) but not necessarily in the lowest energy state, which corresponds to the highest possible density and specific orientation. Extended immersion times (hours to a day) can lead to improved SAM coverage and formation of a highly dense layer, depending on the specific SAM and surface roughness.

In this study, we focused on achieving Nb/Si surface coverage with SAM within a time-frame of minutes, as is typical in SAM patterning^31,32,46^ and other applications^30^. Our primary objective was to suppress oxide growth. We did not observe a measurable effect on relative oxide levels with longer immersion times. Therefore, we limited the SAM immersion time to minutes. This shorter immersion time increases the possibility of defects within the SAM, further exacerbated by the roughness of the film and substrate. Additionally, the sequential application of double-layered SAMs further contributes to the increased surface roughness, as evident from the AFM images. While our surface roughness remains lower than that of the BOE-etched surface, the rapid (seconds-scale) SAM coverage reported in the literature^30,32,46^ is still relevant to our work and is enough to suppress the oxides growth as presented. While surface roughness and packing density can be crucial in certain applications^30,31^, our primary focus was on suppressing oxide growth. Further investigations into SAM properties, particularly within the context of quantum applications, could be a valuable research area.

### Cryogenic setup

**Figure 7:**
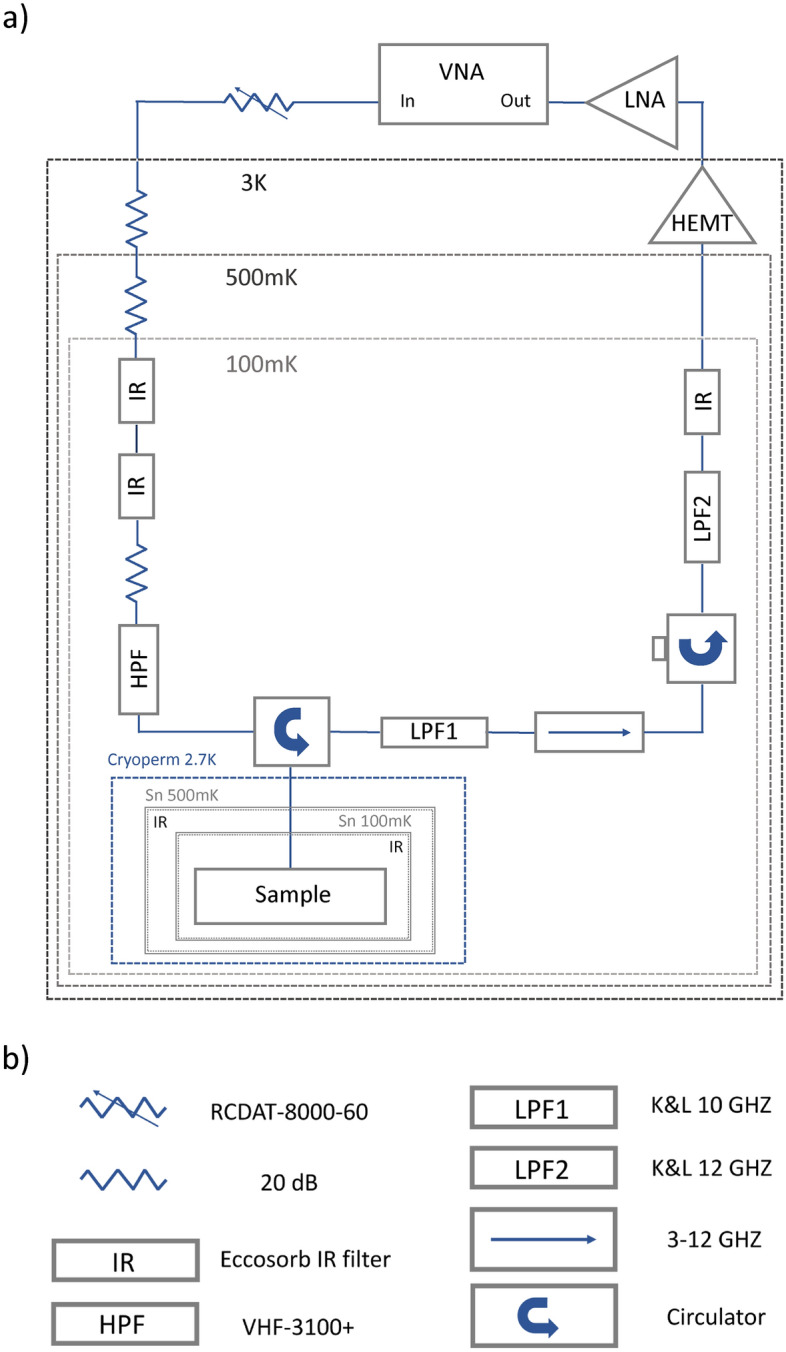
Experimental setup. (**a**) Schematic of the ADR thermal and radiation shielding, and (**b**) microwave components for reflectometry measurements. The resistor symbols represent thermalized attenuators. IR, HPF and LPF are infrared and high-pass and low-pass filters, respectively.

Microwave measurements of devices were performed in HPD Rainer 103 adiabatic demagnetization refrigeration (ADR) cryostat with a base temperature of 100 mK. Devices here were measured in reflection using a microwave circulator with shielding details similar to previous work^25,29^. Resonators devices were wirebonded inside a gold-plated microwave sample box after BOE-SAM process which takes $\sim$ 1 h before loading in ADR that contains two IR and one cryoperm shielding, shown in Fig. 7a, in addition to outer cans of the fridge. Microwave measurements were performed to collect reflectometry scattering parameters, and then quality factors were extracted from S-parameters using Rodhe & Schwartz ZVM vector network analyzer (VNA). Sweep of various excitation powers was performed down to the single-photon regime. The input power was estimated from the VNA output, taking into account the attenuation from a RCDAT-8000-60 programmable attenuator and $\sim$ 100 dB of attenuation (measured at room temperature) from the components inside the cryostat, shown in Fig. 7b.

### Microwave measurements

The performance of a CPW resonator can be characterized by the quality factor, which is a measure of how many oscillations the system needs to dissipate its energy. The internal quality factor $Q_{i}$ is defined as the rate at which the energy is lost to parasitic effects of the environment which indicates how much the device is dissipative (high $Q_{i}$ indicates less dissipation). The coupling quality factor $Q_{c}$ is defined as the rate at which the energy stored in the resonator escapes into a larger component, and in our design $Q_{c}$ indicates how strongly or weakly a resonator is capacitively coupled to the transmission line (fixed in the design $Q_{c} \sim 0.7 \times 10^6$). The two quantities are related by the measured quality factor ($\frac{1}{Q_{r}} = \frac{1}{Q_{c}} + \frac{1}{Q_{i}}$).

Two-level systems (TLS) are a key source of loss in resonators, with their impact expected to reach a maximum at high power levels^13^. As a result, if a TLS channel significantly contribute to the total loss, the intrinsic quality factor ($Q_i$) should rise with power up to a certain point. This behavior helps distinguish TLS-related loss channels from those caused by other mechanisms. The characteristic $Q_i$ values can be determined by changing the power used to probe the resonator and plotting $Q_i$ against the average photon number ($\langle n \rangle$). This relationship can be modeled as follows^48^:

1 $$\begin{aligned} \delta _{int}(T, \langle n \rangle ) = \delta _{\text {TLS}}^0 \frac{\tanh {\left( \frac{hf}{2k_B T} \right) }}{\sqrt{1+(\langle n \rangle /n_c)^\beta }} + \delta _{\text {other}}(T). \end{aligned}$$

where $\delta _{\text {TLS}}^0$ represents TLS loss at low power, $\beta$ (which is less than or equal to 1) is a scaling factor, $n_c$ is the critical photon number, and $\delta _{\text {other}}$ denotes other loss channels that are independent of power and to which $\delta _{int}$ ultimately saturates at high drive powers. In a strong field near a critical photon number $n_{c}$, $\delta _{TLS}$ resonates and gets increasingly coupled to the microwave signal which induces an increase in $\delta _{TLS}$ at low power and temperature, resulting in a decrease of $Q_{i}$. For refection measurements, the average photon number is related to the input power as follows:

2 $$\begin{aligned} \langle n \rangle = \frac{2P_{in}}{\pi h Q_{c} }(\frac{Q_{r}}{f_{0}})^{2} \end{aligned}$$

where $\langle n \rangle$ is the average photon number in the resonator, $P_{in}$ is the drive microwave power and $f_{0}$ is the resonance frequency^49^. For CPW resonators, the electric fields vary along the CPW structure resulting in an effective value of $\beta \sim 0.8$ for spatially uniform TLS distribution, while the value of $\beta = 1$ in uniform structures^50^.

## Data Availability

The datasets generated during and/or analysed during the current study are available from the corresponding author on reasonable request.
